# Multimodal Imaging in Rat Model Recapitulates Alzheimer's Disease Biomarkers Abnormalities

**DOI:** 10.1523/JNEUROSCI.1346-17.2017

**Published:** 2017-12-13

**Authors:** Maxime J. Parent, Eduardo R. Zimmer, Monica Shin, Min Su Kang, Vladimir S. Fonov, Axel Mathieu, Antonio Aliaga, Alexey Kostikov, Sonia Do Carmo, Doris Dea, Judes Poirier, Jean-Paul Soucy, Serge Gauthier, A. Claudio Cuello, Pedro Rosa-Neto

**Affiliations:** ^1^McGill Centre for Studies in Aging, McGill University, Montreal H4H 1R3, Canada,; ^2^Douglas Mental Health University Institute, Montreal H4H 1R3, Canada,; ^3^Department of Biochemistry, Federal University of Rio Grande do Sul, Porto Alegre 90035-000, Brazil,; ^4^Brain Institute (BraIns) of Rio Grande do Sul, Porto Alegre 90610-000, Brazil,; ^5^Montreal Neurological Institute, Montreal H3A 2B4, Canada, and; ^6^Department of Pharmacology and Therapeutics, McGill University, Montreal H3G 1Y6, Canada

**Keywords:** Alzheimer, animal model, biomarkers, imaging, MRI, PET

## Abstract

Imaging biomarkers are frequently proposed as endpoints for clinical trials targeting brain amyloidosis in Alzheimer's disease (AD); however, the specific impact of amyloid-β (Aβ) aggregation on biomarker abnormalities remains elusive in AD. Using the McGill-R-Thy1-APP transgenic rat as a model of selective Aβ pathology, we characterized the longitudinal progression of abnormalities in biomarkers commonly used in AD research. Middle-aged (9–11 months) transgenic animals (both male and female) displayed mild spatial memory impairments and disrupted cingulate network connectivity measured by resting-state fMRI, even in the absence of hypometabolism (measured with PET [^18^F]FDG) or detectable fibrillary amyloidosis (measured with PET [^18^F]NAV4694). At more advanced ages (16–19 months), cognitive deficits progressed in conjunction with resting connectivity abnormalities; furthermore, hypometabolism, Aβ plaque accumulation, reduction of CSF Aβ_1-42_ concentrations, and hippocampal atrophy (structural MRI) were detectable at this stage. The present results emphasize the early impact of Aβ on brain connectivity and support a framework in which persistent Aβ aggregation itself is sufficient to impose memory circuits dysfunction, which propagates to adjacent brain networks at later stages.

**SIGNIFICANCE STATEMENT** The present study proposes a “back translation” of the Alzheimer pathological cascade concept from human to animals. We used the same set of Alzheimer imaging biomarkers typically used in large human cohorts and assessed their progression over time in a transgenic rat model, which allows for a finer spatial resolution not attainable with mice. Using this translational platform, we demonstrated that amyloid-β pathology recapitulates an Alzheimer-like profile of biomarker abnormalities even in the absence of other hallmarks of the disease such as neurofibrillary tangles and widespread neuronal losses.

## Introduction

Alzheimer's disease (AD) is characterized by the accumulation of amyloid-β (Aβ) aggregates in various conformations (Glenner and Wong, 1984; Masters et al., 1985; Dickson, 1997; Selkoe, 2001), the occurrence of neurofibrillary tangles (NFTs) composed of hyperphosphorylated tau proteins (Grundke-Iqbal et al., 1986; Kosik et al., 1988; Goedert et al., 1992), and synaptic dysfunction (Masliah et al., 1989; Mufson et al., 2000).

Considering that all of these pathological manifestations are measurable at least several years before the onset of AD clinical symptoms (Jack et al., 2011; Sperling et al., 2011; Bateman et al., 2012; Buchhave et al., 2012; Jansen et al., 2015), increasing attention has been turned toward biomarkers for AD as measurable proxies of pathophysiological progression, particularly in the presymptomatic stages now referred to as preclinical AD (Dubois et al., 2016). Most notable among those biomarkers is amyloid deposition in the brain as evidenced by positron emission tomography (PET) with radiopharmaceuticals specific to fibrillary amyloid (Klunk et al., 2004) such as [^18^F]NAV4694 (Cselényi et al., 2012) and by the reduction of Aβ concentration in the CSF (Shibata et al., 2000; Strozyk et al., 2003; Deane et al., 2008).

In addition, glucose metabolism as measured by [^18^F]FDG PET and regional brain volumetry from structural MRI (sMRI) both reveal AD-specific regional patterns of synaptic dysfunction and neurodegeneration (Lehéricy et al., 1994; Minoshima et al., 1997; Silverman et al., 2001; Killiany et al., 2002). Finally, resting-state (task-free) functional MRI (rs-fMRI) has been proposed as an indicator of brain connectivity abnormalities likely due to synaptic dysfunctions (Celone et al., 2006; Sperling et al., 2009). There exists an important colocalization of these disruptions with areas of early preferential deposition of amyloid plaques; both tend to be localized to the default mode network (Klunk et al., 2004; Buckner et al., 2008), primarily involving the precuneus and the posterior cingulate, lateral parietal, and medial prefrontal cortices.

The temporal sequence of biomarker abnormality in AD has been modeled extensively using cross-sectional data (Jack et al., 2010; Jack and Holtzman, 2013). What remains unclear, however, is the extent to which this progression can be explained by the vulnerability to Aβ toxicity (Alonso et al., 1994; Jagust, 2016). Biomarker studies in animal models expressing a mutated human amyloid precursor protein (hAPP) gene constitute a powerful platform for addressing such questions. To date, however, the literature on AD imaging biomarkers in transgenic (Tg) animals is composed almost exclusively of research on mice models and has yielded varying and often contradictory results (Kawarabayashi et al., 2001; Bondolfi et al., 2002; Toyama et al., 2005; Maeda et al., 2007; Kuntner et al., 2009; Zimmer et al., 2014). Previously, we showed that this discrepancy is likely due to the diversity of pathological phenotypes across models, the cross-sectional nature of most of these studies, and limited imaging resolution of PET cameras for the mouse brain size (Zimmer et al., 2014).

To test the hypothesis that Aβ aggregation can itself lead to declines in large-scale brain connectivity, metabolism, and cognitive function, we conducted a longitudinal, multimodal biomarker study using the McGill-R-Thy1-APP Tg rat model of AD-like Aβ pathology, which expresses hAPP with Swedish and Indiana mutations. This model displays progressive Aβ aggregation and cognitive deficits, but no NFT inclusions or widespread cell death (Leon et al., 2010; Galeano et al., 2014; Wilson et al., 2017). Considering that rat models have a larger and more complex CNS and higher cognitive abilities compared with mice (Do Carmo and Cuello, 2013), they are particularly advantageous for longitudinal preclinical studies involving imaging modalities (Zimmer et al., 2014). Using the McGill-R-Thy1-APP rat model, we designed a longitudinal, multimodal study for quantifying age-dependent brain Aβ deposition (measured with PET [^18^F]NAV4694 and CSF Aβ_1-42_), progressive synaptic dysfunction (measured with hippocampal volumetry sMRI, rs-fMRI connectivity and PET [^18^F]FDG), and cognitive impairment (measured with a spatial memory task) compared with wild-type (WT) animals.

## Materials and Methods

### 

#### 

##### Procedures.

All procedures described here were performed in accordance with the Canadian Council on Animal Care guidelines and were approved by the McGill University Animal Care Ethics Committee.

##### Experimental design and statistical analysis.

A sample of 26 rats (13 WT Wistar, 13 homozygous McGill-R-Thy1-APP; 7 males and 6 females in each group) was used for this project. Tg McGill-R-Thy1-APP rats (generated and bred at the Cuello laboratory of the Department of Pharmacology and Therapeutics, McGill University) express hAPP751 with the Swedish and Indiana mutations under the control of the murine Thy1.2 promoter, resulting in accumulation of Aβ peptides starting 1 week postnatally, which progress to extracellular plaques at 6–10 months of age in homozygous animals. By 12 months, mature plaques are thioflavin S positive (Leon et al., 2010). Homozygous McGill-R-Thy1-APP also exhibit detectable CSF Aβ38, 39, 40, and 42 species (Iulita et al., 2014), as well as progressive cognitive deficits (Galeano et al., 2014; Qi et al., 2014; Wilson et al., 2017).

All rats were housed at the Douglas Mental Health University Institute animal facility on a 12/12 h light/darkness cycle and had *ad libitum* access to food and water. All animals underwent the procedures described below twice: once at a baseline time point (aged 9–11 months old) and one follow-up (16–19 months); imaging modalities of each animal for each time point were acquired within 2–6 weeks.

##### PET acquisition and processing.

PET acquisition was performed using a CTI Concorde R4 microPET for small animals (Siemens Medical Solutions) and two radiotracers: [^18^F]NAV4694 for imaging Aβ and [^18^F]FDG for imaging glucose metabolism. For [^18^F]NAV4694 scans, anesthesia was first induced using 5% isoflurane in 0.5 l/min oxygen and then maintained throughout the procedure with 2% isoflurane. A 9 min transmission scan using a rotating [^57^Co] point source was followed by a bolus injection of the radiotracer in the tail vein (13.3 ± 0.9 MBq in 200 μl, with a specific activity of 85.97 ± 46.47 GBq/μmol), concomitant with the beginning of the emission scan, which lasted for 60 min in list mode. The data were then reframed into 27 sequential time frames of increasing durations (8 × 30 s, 6 × 1 min, 5 × 2 min, and 8 × 5 min). For [^18^F]FDG, tracer injection was done in the tail vein of awake animals (12.7 ± 1.1 MBq in 200 μl), which were anesthetized (5% isoflurane in 0.5l/min oxygen for induction, reduced to 2% during the scan) 50 min later to perform a 20 min emission scan (in a single static time frame) and a 9 min transmission scan. Breathing rate was monitored throughout both scanning procedures; temperature was monitored using a rectal thermometer and maintained at 37 ± 1°C using an electric blanket. Images for both tracers were reconstructed using a maximum *a posteriori* (MAP) algorithm (voxel size: 0.6 · 0.6 · 1.2 mm) and corrected for scatter, dead time, and decay.

**Figure 1.**
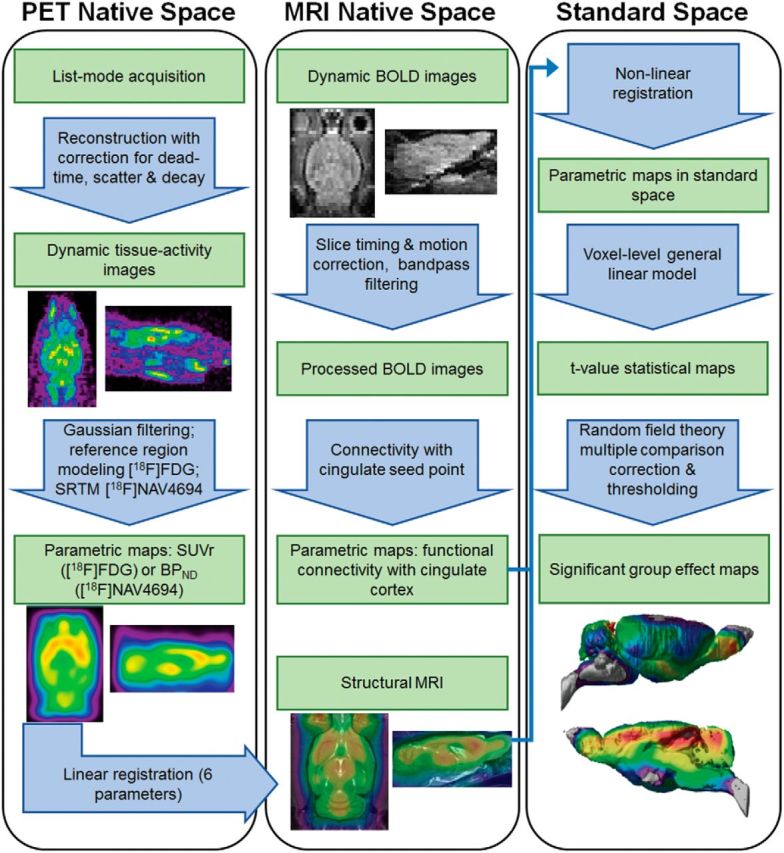
Processing and analytical pipeline for imaging data. PET data were acquired in list mode and then reconstructed with correction for dead time, scatter, and decay. Resulting tissue activity images were filtered with a Gaussian kernel and parametric maps were generated for each radiotracer and then linearly registered to their respective structural (FISP) MRI. For rs-fMRI, dynamic resting-state images were corrected for slice timing and motion and then band-pass filtered. Resulting processed images were correlated at the voxel level to a seed point in the cingulate cortex to generate parametric maps of connectivity. Parametric maps of connectivity, SUVr, and BP_ND_ in individual MRI space were then nonlinearly coregistered to a standard space consisting of an averaged sMRI. Group effects were assessed with a voxel-level linear model corrected for multiple comparisons using the random field theory approach. SRTM, Simplified reference tissue method.

MINC tools (www.bic.mni.mcgill.ca/ServicesSoftware) were used for image processing and analysis. Image processing steps are summarized in Figure 1. Briefly, parametric maps were generated. For [^18^F]NAV4694, the binding potential (BP_ND_) was calculated for each voxel using the simplified reference tissue method at the voxel-level (Gunn et al., 1997) with cerebellar gray matter as a reference region. For [^18^F]FDG, standardized uptake value ratio (SUVr) images were generated by normalizing the tissue radioactivity image using the pons as a reference tissue. Each resulting parametric image was first coregistered to the individual animal's sMRI (see below) using six degrees of freedom (rigid body transformation), then nonlinearly transformed to a standardized rat brain space created from the WT Wistar rats used in the present study to account for differences in brain morphology.

##### MRI acquisition and processing.

MRI acquisition was performed in a 7 T BioSpec 70/30 USR dedicated animal MRI (Bruker) equipped with Avance III electronics and the 500V/300A B-GA12S2 gradient upgrade with a standard 40 mm quadrature volumetric transceiver. Animals were anesthetized with a 1% isoflurane/medical air mixture. A constant 37°C air flow was used to keep the animals warm.

Structural imaging was obtained using the Bruker standard 3D-True Fast Imaging with Steady State Precession pulse sequence (3D-TrueFISP, a balanced Steady State Free Precession type sequence). To remove banding artifacts, a root-mean-square image of eight phase advance (angles of 0–315 degrees in increments of 45) acquisitions was obtained. Each TrueFISP phase angle acquisition was acquired as follows: slices oriented in the rostrocaudal axis, FOV of 36 × 36 × 36 mm with a matrix of 180 × 180 × 180, TE/TR of 2.5/5.0 ms, NEX of 2, flip angle of 30°, and a bandwidth of 50 kHz; no accelerations were used. The resulting image is an average of 16 acquisitions with an isotropic 200 μm resolution and was acquired in a total scanning time of 46 min 30 s. Hippocampal volumes were measured using manual segmentation performed by an experimenter blinded to the group conditions and normalized by the intracranial volume.

The rs-fMRI acquisitions were completed immediately after the anatomical scans using the standard Bruker 2D-Spin Echo, Echo Planar pulse sequence (2D-SE-EPI) and the following parameters: slices oriented in the rostrocaudal axis, FOV of 25.6 × 25.6 mm with a matrix of 64 × 64 and 32 slices of 1.0 mm for a final resolution of 400 × 400 × 1000 μm, interslice distance of 1.0 mm, TE/TR of 15/2000 ms, flip angle of 70°, bandwidth of 300 kHz, 4 dummy scans to establish steady state, and 450 repetitions for a total scan time of 15 min. A partial-FT acceleration factor of 1.34 (16 overscans) was used, with standard fat suppression and 5 standard saturation slices to isolate the brain volume; the fifth saturation band was used over highly fatty throat areas. Finally, the standard EPI navigator was used, along with automatic ghost correction and automatic trajectory adjustment.

The first four volumes (8 s) were discarded to account for transient drift. Using AFNI (https://afni.nimh.nih.gov), the dynamic functional images were corrected for slice time and motion and then band-pass filtered between 0.01 and 0.15 Hz. Connectivity maps were generated by correlating with a seed point in the cingulate cortex, a component of the rat's default mode network (Lu et al., 2012), which has been shown in this model to be vulnerable to fibrillary Aβ accumulation (Parent et al., 2013). Resulting images were nonlinearly transformed to the standardized rat brain space.

##### Spatial memory.

The spatial memory of each rat was assessed using the Morris water maze (MWM) (Morris, 1984) over 4 consecutive days, with 4 trials/d, a maximum trial length of 90 s (rats were placed on the platform after unsuccessful 90 s trials), and 1 h between each trial. Each trial was started in a different quadrant and external cues were placed outside the pool for navigation. The time to find the platform was measured automatically using overhead camera tracking with ANY-maze video-tracking software (Stoelting) and used as an outcome measure. One hour after the last trial on the fourth day, one probe (no platform) trial to assess reference memory and one visible platform trial was also conducted to account for swim speed and gross visual deficits.

##### CSF sampling.

Under 5% isoflurane anesthesia, 100–150 μl of CSF was collected from each rat through direct puncture of the cisterna magna. Concentrations of Aβ1-42 in the CSF samples were measured using a multiplex xMAP Luminex platform with the ELISA kit INNOTEST β-AMYLOID_1-42_ (Fujirebio Europe), with calibrators from 62.5–4000 pg/ml.

##### Immunohistochemistry.

After completion of the second time point experiments, rats were anesthetized using equithesin (pentobarbitol based, 2.5 ml/kg, i.p.) before transcardiac perfusion with cold 0.1 m phosphate buffer (PB), pH 7.4. The brains were removed, divided into hemispheres, postfixed in 4% paraformaldehyde in 0.1 m PB for 24 h at 4°C, and then equilibrated in a solution of 30% sucrose in 0.1 m PB. Coronal sections of 40 μm thickness were obtained using a freezing sledge microtome (SM 2000R; Leica). Free-floating sections (3 per animal) were collected in PBS containing 10 mm Na_2_HPO_4_, 150 mm NaCl, and 2.7 mm KCl and processed for immunohistochemistry. Brain sections were incubated first in McSA1 (MediMabs), a mouse monoclonal antibody detecting human Aβ (Grant et al., 2000), and then in goat anti-mouse antibody (MP Biochemicals), followed by a mouse anti-peroxidase monoclonal antibody complex (MAP/HRP complex; MediMabs), and developed using 3,3′-diaminobenzidine as the chromogen (Vector Laboratories). Images of Aβ immunoreactivity were acquired using a Zeiss microscope equipped with an AxioCam HRc digital camera (Carl Zeiss) and Axiovision 4.8 software.

##### Statistical analyses.

For imaging outcome measures ([^18^F]NAV4694, [^18^F]FDG and rs-fMRI), group effects were estimated using a voxel-level general linear model. Resulting *t*-statistical maps were corrected for multiple comparisons using a random field theory-based approach (Worsley et al., 1996) for an adjusted threshold of *p* < 0.05 in clusters of at least 30 mm^3^. Longitudinal changes in [^18^F]NAV4694 binding and [^18^F]FDG uptake were measured with the voxel-level differences between baseline and follow-up parametric images, normalized by the baseline, and expressed as maps of average percentage changes.

## Results

No significant effect of sex was found for any measurement; therefore, males and females were grouped together for all subsequent analyses.

### Aβ accumulation induces time-dependent changes in glucose metabolism and connectivity

**Figure 2.**
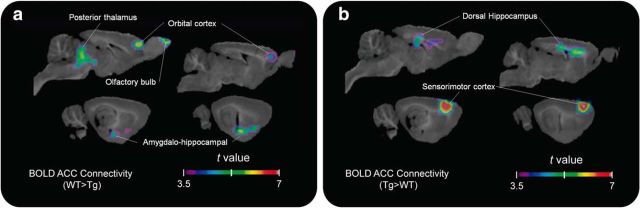
Early functional connectivity disruption in Tg animals. *t*-statistical rs-fMRI connectivity group contrasts (*n* = 13 animals per group) at baseline are shown overlaid on a template structural image (sagittal slices 1–4 mm lateral to midline at 1 mm intervals). ***a***, ***b***, Connectivity with the cingulate seed point is reduced for Tg rats in the orbital cortex, thalamus, and amygdalohippocampal area (***a***), and upregulated in the dorsal hippocampus and primary sensory and motor cortical regions (***b***). Clusters of at least 30 mm^3^ are shown for values corresponding to *p* < 0.05 after multiple-comparisons correction.

We first examined the consequences of incremental aggregation of Aβ in the McGill-R-Thy1-APP Tg rat model of AD-like Aβ pathology by PET and rs-fMRI. Although some fibrillary Aβ is visible by immunohistochemistry at the baseline time point (9–11 months), especially in the dorsal hippocampus (see Fig. 6*b*), no significant group difference in [^18^F]NAV4694 binding was found at that age. Similarly, glucose metabolism as measured by [^18^F]FDG uptake was not significantly altered in younger Tg animals. Group contrast for rs-fMRI showed clusters of significantly lower cingulate connectivity in the Tg group centered on the orbital cortex and the thalamus (*k* = 34.74 mm^3^, peak *t*_(24)_ = 6.528, *p* < 0.0001), as well as higher connectivity with the dorsal hippocampus and sensorimotor cortical areas (Fig. 2).

**Figure 3.**
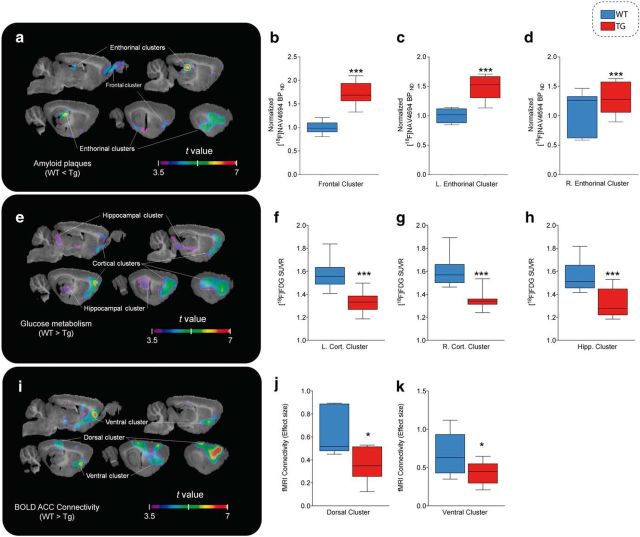
Amyloid plaques, glucose hypometabolism, and functional connectivity impairments in Tg animals. *t*-statistical group contrasts at follow-up time point are shown overlaid on a template structural image. Significant group contrasts after multiple comparison correction in clusters of at least 30 mm^3^ are projected on sagittal slices (1–5 mm lateral from midline at 1 mm intervals). Box-and-whisker plots show the distribution of the parametric measures for each cluster in WT (blue) and Tg (red) animals. ***a***–***d*** [^18^F]NAV4694 BP_ND_ (*n* = 10 Tg, 9 WT; ***a***) is significantly higher for Tg rats in the olfactory bulb and frontal cortex (***b***) and in the left (***c***) and right (***d***) dorsal hippocampus and the entorhinal and insular cortices. ***e***–***h***, [^18^F]FDG uptake (*n* = 10 Tg, 9 WT; ***e***) is lower in Tg rats across the left (***f***) and right (***g***) frontal cortex and in the genu of the hippocampus, ventral thalamic nuclei, and colliculi (***h***). ***i***–***k***, rs-fMRI (*n* = 8 Tg, 8 WT; ***i***) connectivity is weaker in dorsal hippocampus and the retrosplenial cortex (***j***) as well as in prelimbic areas and the basal forebrain (***k***). ***p < 0.0005; *p < 0.05.

At the follow-up time point (16–19 months of age), all three imaging outcomes showed significant group differences (Fig. 3). For PET Aβ load, the Tg group had significantly higher binding in a cluster covering the olfactory bulb and the infralimbic cortex and spreading laterally to the insular, perirhinal, and entorhinal cortices (*k* = 113.34 mm^3^, peak *t*_(17)_ = 6.659, *p* < 0.0001), in which the ratio of BP_ND_ in the Tg group compared with the nonspecific binding observed in WT animals was 1.734 ± 0.428. A second cluster covering the dorsal hippocampi, the caudal piriform cortex, and amygdala (*k* = 70.24 mm^3^, peak *t*_(17)_ = 7.854, *p* < 0.0001) showed an average ratio of 1.767 ± 0.467 (Fig. 3*a–d*).

Group contrast of [^18^F]FDG PET hypometabolism revealed 2 symmetrical clusters of significant differences where the WT group had higher uptake than Tg animals (*t* > 3.58), located in the ventral orbital, secondary motor, cingulate, prelimbic, barrel, and entorhinal cortices (left hemisphere: *k* = 133.09, peak *t*_(17)_ = 6.46, *p* < 0.0001; average SUVr of 1.449 ± 0.157 for WT and 1.343 ± 0.14 for Tg; right hemisphere: *k* = 117.48 mm^3^, peak *t*_(17)_ = 6.403, *p* < 0.0001; SUVr of 1.484 ± 0.123 for WT and 1.369 ± 0.099 for Tg). A third, median cluster covered the ventral thalamus and medial geniculate, as well as the hippocampal genus and the inferior colliculi (*k* = 82.36 mm^3^, peak *t*_(17)_ = 4.417, *p* = 0.0004), where the WT group had an average SUVr of 1.448 ± 0.121 compared with 1.343 ± 0.112 for the Tg group (Fig. 3*e–h*).

Connectivity with the cingulate seed point, as measured by rs-fMRI, was lower in Tg animals, including the prelimbic and infralimbic cortices, basal forebrain, ventral caudate putamen, dorsal hippocampi, parietal association cortex, and endopiriform nucleus (*k* = 242.442 mm^3^, peak *t*_(15)_ = 7.392, *p* < 0.0001) (Fig. 3*i–k*).

**Figure 4.**
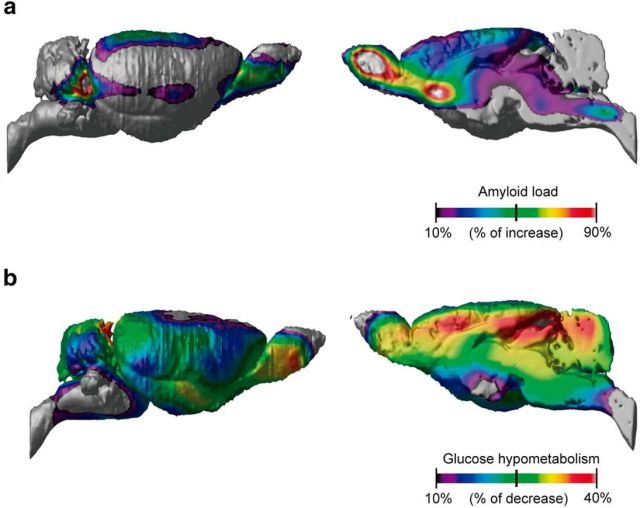
Regional progression of amyloid plaques and glucose hypometabolism over time. Percentage change values overlaid on a brain surface and midsagittal projections. Longitudinal progression of PET biomarkers in Tg animals over a 6 month period (*n* = 10 animals with two time point measurements per biomarker) is shown. ***a***, [^18^F]NAV4694 binding increase over time is most prominent in the parietal and entorhinal cortices and in the basal forebrain and olfactory bulb. ***b***, [^18^F]FDG uptake longitudinal decreases are present throughout the encephalon, with the highest changes across the cortical mantle and in the cerebellum.

In terms of age effect, the Tg but not the WT group showed a progressive increase of fibrillary Aβ, with [^18^F]NAV4694 BP_ND_ reaching differences of up to 47.44% in the parietal association and retrosplenial cortices, 91.22% in the caudal entorhinal cortex, and 94.08% in the basal forebrain and olfactory bulb (Fig. 4*a*). Conversely, [^18^F]FDG uptake decreases (Fig. 4*b*) were observed throughout the brain in Tg animals, with the highest reductions located in frontal (38%) and parietal (37%) cortices as well as the cerebellum (36%). Together, these findings suggest that Aβ deposition per se is sufficient to cause abnormalities in glucose metabolism and connectivity.

### Aβ-induced functional deficits are reflected by spatial memory impairments

**Figure 5.**
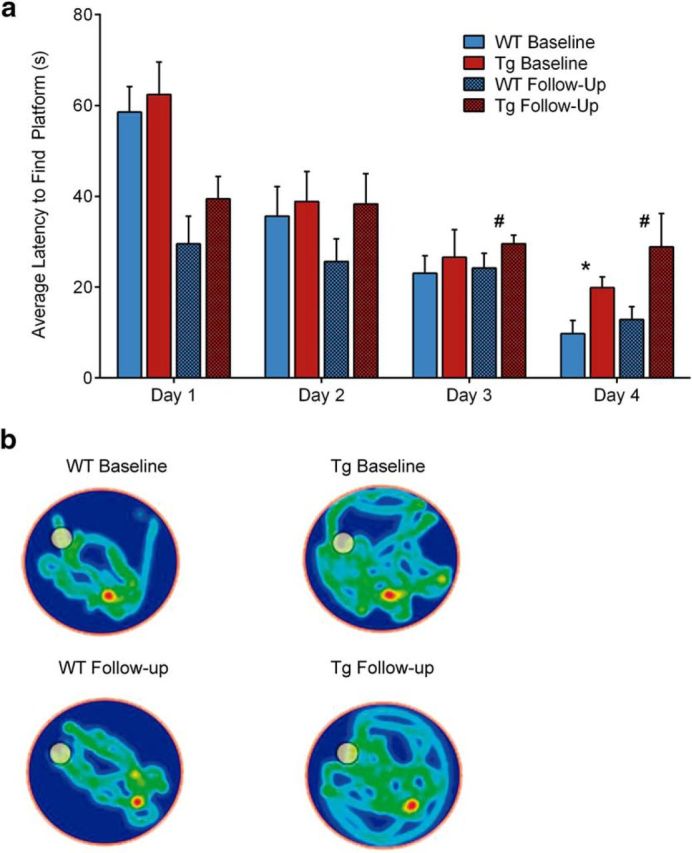
Spatial memory deficits in Tg animals. ***a***, Average time to find platform during the learning phase of the MWM yask. Significant group effects were found at day 4 for baseline measurements (*) and for days 3 and 4 at follow-up (**#**). In addition, a long-term learning effect is visible through improved day 1 performance at follow-up compared with baseline. Error bars represent mean ± SEM of each group (*n* = 13 per group at baseline; 11 Tg and 9 WT animals at follow-up) with 4 repetitions per animal per day. ***b***, Representative occupancy plots of one day 4 trial for each group and time point. Circle denotes starting position.

Spatial memory, as tested using the MWM task, showed a learning effect for both groups at both time points, shown as the latency to locate the platform in the learning phase of the task from days 1–4 (Fig. 5). For the baseline time point, there was a significant genotype effect only on the fourth day of testing, with the WT taking significantly less time to locate the platform than the Tg animals (*F* = 8.996, *p* = 0.007). At follow-up, the WT group performed significantly better than Tg for both the third (*F* = 5.903, *p* = 0.028) and fourth (*F* = 5.352, *p* = 0.038) days of learning. In addition, both groups performed significantly better on the first day of testing at follow-up than they did at baseline (*F* = 8.243, *p* = 0.001). Despite the differences during the learning phase, both groups reached comparable performance during the probe trial as measured by proportion of time spent in the target quadrant (WT: 38.9 ± 14.9%; Tg: 39.9 ± 9.5%). Latency to reach the platform during a trial where the platform was visible did not differ significantly between Tg (17 ± 10.4 s) and WT (14.4 ± 5.2 s). These findings illustrate observable cognitive impairments as a consequence of Aβ-induced metabolic and synaptic dysfunctions.

### Aβ deposition in the brain is reflected by decreased Aβ CSF levels and brain volume

**Figure 6.**
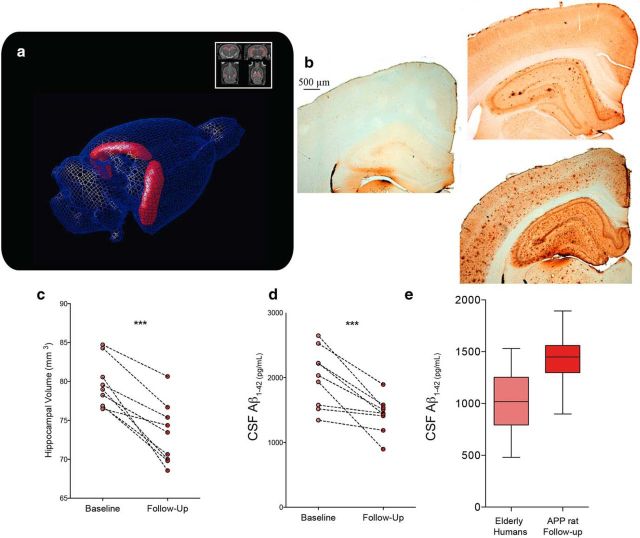
Hippocampal volumetry and CSF Aβ1-42 concentrations. ***a***, ***c***, Volumetric analysis of the hippocampus after manual segmentation showing average decrease of normalized volume from 79.606 to 73.283 mm^3^ (7.94% lower) in Tg animals, with individual trajectories (*n* = 8 animals with two time point measurements) of hippocampal volume between baseline and follow-up. ***b***, Aβ immunoreactivity detection using McSA1 antibody in the brain tissue of representative WT (left) and Tg animals at 11 (top right) and 20 months of age (bottom right). Slices are 3.3 mm posterior from bregma, showing the dorsal hippocampus and the retrosplenial, somatosensory, and auditory cortices. ***d***, Individual trajectories (*n* = 8 animals with 2 time point measurements) of CSF concentrations of Aβ1-42 between baseline and follow-up for Tg rats. Concentrations went from an average of 2002.507 to 1433.031 pg/ml, representing a 28.44% decrease. ***e***, Distribution of CSF concentrations of Aβ1-42 in elderly humans (*n* = 62, 75.5 ± 6.8 years old) compared with those of Tg rats at the follow-up time point (*n* = 8). ***p < 0.0005.

Last, two other common AD biomarkers, hippocampal volumetry and CSF Aβ1-42, were studied using the McGill-R-Thy1-APP model. The normalized hippocampal volumes of Tg animals decreased from 79.606 ± 3.093 mm^3^ at baseline to 73.283 ± 3.93 at follow-up (paired *t*_(8)_ = 6.328, p = 0.0002), whereas there was no significant age effect for WT rats (Fig. 6*a*,*c*). In addition, the CSF Aβ1-42 concentrations in older Tg rats decreased by 28.44% compared with the first time point assay (Fig. 6*d*), from 2002.507 ± 453.008 pg/ml at baseline to 1433.031 ± 273.349 pg/ml at follow-up (paired *t*_(8)_ = 4.513, *p* = 0.002), with overall concentrations being comparable to those observed in elderly human cohorts (Fig. 6*e*).

## Discussion

In summary, we have shown in this longitudinal study that Aβ aggregates secondary to the expression of mutated hAPP in the rat brain (devoid of NFTs or widespread neuronal depletion) are sufficient to cause specific brain injury quantifiable using the same biomarkers that are used as outcome measures in AD clinical studies. In aged McGill-R-Thy1-APP rats, amyloidosis was observed by increased [^18^F]NAV4694 binding in addition to decreased CSF Aβ_1-42_ concentrations. Compared with WT controls, the Tg animals showed progressive functional decline, reflected by reduced resting brain connectivity and glucose metabolism, as well as spatial memory impairments measured by the MWM task.

Interestingly, both rs-fMRI and behavioral measures showed abnormalities before mature fibrillary plaques or glucose hypometabolism were detectable by microPET imaging. These results support the notion that human Aβ oligomeric aggregates exert toxic effects before the formation of mature, thioflavin-positive Aβ plaques (Walsh et al., 2002; Forny-Germano et al., 2014). Indeed, previous electrophysiological studies conducted in the McGill-R-Thy1-APP and other models of human brain amyloidosis conducted at this disease stage suggest that early brain connectivity changes or memory declines observed in our cohort are conceivably functional consequences of synaptic alterations (Iulita et al., 2014; Qi et al., 2014; Wilson et al., 2017).

As suggested previously, adaptations of network architecture such as recruitment and strengthening or weakening of specific connections might occur as a consequence of Aβ aggregates (Greicius et al., 2004; Buckner et al., 2009; Gardini et al., 2015). In fact, the brain network abnormalities reported here possibly represent a large-scale signature of Aβ-induced synaptic dysfunction rather than disruption of the underlying structural connections (Lacor et al., 2007; Bao et al., 2012). Remarkably, the fact that human Aβ aggregates enhance the connectivity between hippocampus and cingulate cortex in the animal model and in mildly cognitively impaired patients indicates susceptibility of this specific memory network component to Aβ (Bai et al., 2009; Elman et al., 2014; Gardini et al., 2015). In fact, early functional deficits preceding the onset of fibrillary Aβ are possibly related to synaptic vulnerabilities, which supports the concept that localized Aβ deposition may be dependent on the default patterns of activity preceding disease onset (Buckner et al., 2005). It should be noted that, although these early functional changes underscore deleterious effects of pre-plaque Aβ aggregates, we cannot discard the possibility that further damage is imposed by fibrillary Aβ deposits in later disease stages.

Although the progression rates of biomarker abnormalities over time vary significantly throughout the brain, both amyloidosis and hypometabolism are contained within a range of 20–40% in the frontoparietal areas between the cingulate and retrosplenial cortices. Specifically, in the parietal association and retrosplenial cortices, fibrillary Aβ deposition increased by an average of 34% over the 6 month period separating baseline and follow-up scans, whereas glucose metabolism decreased by an average of 31%. This characteristic glucose hypometabolism indicates synaptic dysfunction, which can be attributed both to neuronal or astrocytic dysfunction (Zimmer et al. 2017).

Colocalization among Aβ fibrillary deposition, hypometabolism, and connectivity decline occurred in the vicinity of the rhinal fissure encompassing the somatosensory and limbic cortices, whereas other brain regions showed partial biomarker abnormality overlapping. For instance, both fibrillary Aβ and connectivity impairments converge in cortical and hippocampal regions, which confirms that Aβ is sufficient to predict functional connectivity decreases in resting-state networks and in the hippocampal formation in humans (Hedden et al., 2009). Conversely, whereas progressive accumulation of fibrillary Aβ was most prominent in the olfactory bulb and basal forebrain, decreases in glucose uptake was largest in the cortex. This regional dissociation likely reflects a selective vulnerability of these cortical areas to Aβ toxicity or could be explained by a downregulation of the basalo-cortical projections. Indeed, regional declines in metabolism measurable with [^18^F]FDG PET can be induced by an injury in a remote brain region (Meguro et al., 1999).

The basal Aβ_1-42_ levels observed in these animals are comparable to those in human populations (Fig. 6*d*), which underlines the translational value of the present observations. Mild brain atrophy measured with sMRI indicated a modest but significant effect of Aβ on volumetry limited to the hippocampus, which is the only structure where cell death is observed in McGill-R-Thy1-APP rats (Heggland et al., 2015). Resilience of the surrounding cortical areas to atrophy might be explained by the absence of NFTs because native murine hyperphosphorylated tau is not prone to aggregation. Alternatively, the follow-up time point of this study may not have been late enough for the initiation of more pronounced brain atrophy. Future studies of this model will investigate CSF levels of hyperphosphorylated tau to gain a better understanding of the progression of pre-atrophy neurodegeneration. Finally, the early memory impairment observed here seems to contrast with the human sequence of biomarkers abnormalities modeled by Jack and Holtzman (2013), which could be explained by an absence of neural reserve in the less evolved rodent CNS. Learning deficits were also observed for a visual association task in this animal model (Wilson et al., 2017), whereas hemizygous Tg animals (±) showed impairments in working memory as early as 6 months of age (Galeano et al., 2014).

Our findings indicate that longitudinal biomarker acquisitions in rodents recapitulate large-scale observational and interventional studies in humans, specifically in prodromal and early stages of the disease. This is the first longitudinal, multiparametric study using a robust rat model of Aβ pathology illustrating progressive abnormalities in AD biomarkers. With only a single transgene insertion site per allele, this rat model has minimal genetic invasion compared with other animal models, yet was able to reproduce a biomarker profile closely analogous to that of human disease. Based on the present observations, we propose that biomarker abnormalities as a function of Aβ pathology are more evident at the level of large-scale brain network connectivity and regional brain metabolism measurements than at that of brain atrophy or memory impairment measurements.
